# A Remarkable Remission: Treating HMA Refractory Transforming MDS with Single-Agent Low-Dose Cytarabine Leading to an Ongoing Six-Year OS

**DOI:** 10.1155/2020/1540370

**Published:** 2020-02-14

**Authors:** David Palmer, Lydia Jones

**Affiliations:** Epsom and St. Helier University Hospitals NHS Trust, Dorking Road, Epsom KT187EG, UK

## Abstract

Hypomethylating agents (HMA) are the standard of care for patients ≥65 years with intermediate-high risk myelodysplastic syndrome (MDS) unsuitable for intensive therapy or stem cell transplant (SCT). However, many patients will develop relapse/refractory disease, at which point limited treatment options remain. There has been a lot of research into investigational agents following HMA failure, especially now into targeted therapy, but there is no final consensus or convincing data to guide clinicians. Low-dose cytarabine (LDAC) has been in the armamentarium for some time, but the value of LDAC is judged differently by various guidelines. Nevertheless, in a subgroup of patients who fail on a HMA and wish to continue treatment, LDAC may still have the potential to improve overall survival (OS). In this case report, we present an 85-year-old gentleman with HMA refractory high-risk/transforming MDS (with a noncomplex karyotype) achieving an ongoing six-year OS with single-agent second-line LDAC. LDAC may therefore still be considered by clinicians as a therapeutic option, but when available, patients should be enrolled on a clinical trial.

## 1. Introduction

LDAC as a single-agent has been accepted to provide a modest benefit with a median OS of ≤5 months in high-risk MDS patients who progress to acute myeloid leukaemia (AML) [1]. It has previously been stated that LDAC might be considered for high-risk patients that are not candidates for any intensive treatment/where an HMA is not feasible [2], whereas others do not recommend the use of LDAC [3]. Another large multicentre RCT did not demonstrate any improved OS with LDAC [4]. More recently, Santini has summarised the potential therapeutic options that exist after a HMA has failed [5].

## 2. Case Report

We present the case of an 85-year-old gentleman with a past medical history of a malignant melanoma of the leg in his mid-thirties which was treated with excision and intralymphatic radioactive phosphorous injections. Over time, this resulted in intractable lymphedema. In his late seventies, he suffered with recurrent bouts of cellulitis of this leg which eventually led to the presence of a bicytopaenia being discovered on a full blood count. He was referred to haematology where his counts were further monitored; however, progressive cytopaenias eventually led to a bone marrow aspirate and trephine (BMAT). The presence of 17% blasts on BMAT gave a preliminary diagnosis of transforming MDS. His counts at diagnosis for haemoglobin, platelets, and absolute neutrophil count (ANC) were 135 g/dL, 66 × 10³/*μ*L and 0.8 × 10³/*μ*L, respectively. He was tP53 wild type, but no further information was known about any other genetic mutations at the time of diagnosis. Cytogenetic analysis showed -Y karyotype. His subtype was MDS with excess blasts 2. Based on these features, the revised international prognostic scoring system (IPSS-R) score at diagnosis was 4.0 classifying him as intermediate risk with a median survival of three years with treatment. His ECOG performance status (PS) was 1. This gentleman was keen to commence treatment as he expressed a wish to remain ambulatory and independent of transfusions as possible.

He was commenced on the HMA Azacytidine in 2012 and completed twelve cycles in total before a repeat BMAT had shown a transformation to AML with a blast count of 28%. His blood counts at this time had further worsened (Hb 97 g/dL, platelets 12 × 10³/*μ*L and ANC 0.6 × 10³/*μ*L) requiring platelet transfusion support. He had now progressed into the IPSS-R high-risk category. He expressed a wish to continue with treatment as he had an important life event coming up. His PS at this time remained 1. In 2013, he was switched to LDAC. He completed 3 cycles of induction therapy (20 mg subcutaneously BD for 7 days) and then a further 3 cycles of consolidation therapy (20 mg subcutaneously BD 5 days a week). A repeat BMAT had shown a blast reduction from 28% to 4% and a good recovery of normal marrow. His ANC had increased to >1.0 and platelets >100 × 10³/*μ*L. He had therefore met the criteria for a complete hematologic remission (CR).

Since this gentleman's initial response to LDAC in 2013, he has now completed 42 cycles (with 8 week intervals every 12 cycles). However, due to intermittent severe neutropenia (ctCAE ≥3) over the last six years, he has had sixteen significant hospital admissions due to infections. This has frequently led to treatment delays and he has often required GCSF as a therapeutic adjunct. Still, he has had a remarkable ongoing OS of six years with minimal transfusion burden and, despite numerous inpatient admissions, tolerable toxicities.

## 3. Discussion

The incidence of MDS in the general population is 5/100,000, increasing to 40/100,000 among subjects aged >70 years; making it the most frequent haematological disease in the elderly [6]. For these patients with intermediate-high risk MDS, the HMA Azacytidine is the only NICE approved agent [7]. However, in many older patients, Azacytidine is not so well tolerated. One such study reports a discontinuation rate of 32% after the first cycle, with 69% of patients not reaching the targeted number of cycles [8]. As a result, many older patients will discontinue treatment or develop relapse/refractory disease. This underscores the need for second line agents in this cohort. LDAC has been used in various schedules for several years in AML and high risk MDS. It has previously been proposed that the sequential use of LDAC after HMAs is able to provide an enhanced anticancer effect due to epigenetic remodelling which further sensitizes leukaemic cells to subsequent cytotoxic therapy [9].

Whilst the patient in this case had transforming high risk MDS, he did have very favourable cytogenetics. In a 2007 study patients with high risk MDS/AML were randomised to receive either LDAC or hydroxycarbamide [10]. Regardless of treatment arm median OS was significantly reduced by the presence of adverse cytogenetics and all the patients randomised to the LDAC arm had an OS <1 year. Of the patients with favourable/intermediate cytogenetics there was no difference in outcomes between ages, with patients >75 deriving a similar benefit to those <75. This therefore demonstrates the importance of cytogenetics in independently prognosticating a response to LDAC. Another study has previously looked at elderly patients with AML unsuitable for intensive chemotherapy and randomised them to supportive care, LDAC, or conservative chemotherapy [11]. Patient karyotyping was excluded from this study allowing the authors to isolate a number of other prognostic factors on multivariate analysis, independent of treatment. Notably if older patients (>75) had ≥2 of any of the following features; PS ≥2, platelet count <50 × 10³/*μ*L and a peripheral blast count of >5.0 × 10³/*μ*L, they had a significantly worse median OS when compared to younger patients also with ≥2 of the above features. This was 41 and 130 days, respectively. These studies described demonstrate that predicting a response to LDAC in older patients with high risk MDS is complex and influenced by multiple factors.

The patient presented in this case suffered with significant neutropenia leading to frequent hospitalisation. In a previous study toxicity profiles between Azacytidine and conventional regimes (including LDAC) in patients with high risk MDS/AML were compared [12]. Whilst the incidence of neutropenia and febrile neutropenia were significantly higher in the LDAC arm compared to Azacytidine, grade 3/4 adverse events occurred with similar frequency (neutropenia, 22.9% compared to 19.9%, and febrile neutropenia, 24.8% compared to 25%, respectively). However, the time spent in hospital for adverse events was lower in the Azacytidine group compared to the conventional regimes group which were 28.5 days and 38.3 days, respectively, per patient year of drug exposure (*p* < 0.0001).

Many trials to date have looked at combined therapies with LDAC. The use of Clofarabine (combined with LDAC) in intermediate-high-risk patients who had failed after ≥4 cycles of a HMA led to a median OS of 11 months [13]. The use of CHG (LDAC, Homoharringtonine, and GCSF) matched CR rates to the HMA Decitabine in high-risk MDS, though this was not the case for patients with complex karyotypes [14]. The hedgehog inhibitor Glasdegib has been shown to provide a median OS benefit of 3.9 months when combined with LDAC and compared to LDAC monotherapy in previously untreated high-risk MDS/AML patients [15]. Again this study demonstrated no survival advantage in patients with adverse cytogenetics. Other studies have been less promising. A phase I/II trial using combination LDAC and Sorafenib in patients >60 with previously untreated high-risk MDS/AML with FLT3 mutations did not meet the prespecified criteria to complete the second phase II stage due to limited activity [16]. As a result, there is still no consensus on whether LDAC should be used as a monotherapy or in combination.

In summary, previous studies have shown varying results from LDAC in patients with HMA refractory high-risk/transforming MDS, many of these more recently demonstrating no significant improvement in OS. The patient presented in this case had a very favourable karyotype and cytogenetics is clearly important in predicting a response to LDAC. However, it is important clinicians are sensitive to other factors such as age and PS as well. In the future, the treatment of transforming MDS/AML is likely to move further towards targeted therapy. However, outside of a clinical trial, carefully selected older patients with HMA refractory disease and noncomplex karyotypes may still have the potential to have a response to LDAC, perhaps due to its sequential effect after a HMA, as was demonstrated in this case.
